# Risk Factors for Subdural Haematoma in Patients with Spontaneous Intracranial Hypotension

**DOI:** 10.1371/journal.pone.0123616

**Published:** 2015-04-08

**Authors:** Ping Xia, Xing-Yue Hu, Jin Wang, Bei-Bei Hu, Qing-Lin Xu, Zhi-Jie Zhou, Min Lou

**Affiliations:** 1 Department of Neurology, Sir Run Run Shaw Hospital, Zhejiang University School of Medicine, 3 East Qingchun Road, Hangzhou, 310016, China; 2 Sir Run Run Shaw Institute of Clinical Medicine of Zhejiang University, 3 East Qingchun Road, Hangzhou, 310016, China; 3 Department of Orthopaedic Surgery, Sir Run Run Shaw Hospital, Zhejiang University School of Medicine, 3 East Qingchun Road, Hangzhou, 310016, China; 4 Department of Neurology, 2nd Affiliated Hospital, Zhejiang University School of Medicine, 88 Jiefang Road, Hangzhou, 310009, China; Duke University Medical Center, UNITED STATES

## Abstract

Subdural haematoma (SDH) is a potentially life-threatening complication in patients with spontaneous intracranial hypotension (SIH). In serious cases, SIH patients who present with SDHs develop neurological deficits, a decreased level of consciousness, or cerebral herniation, and may even require an urgent neurosurgical drainage. Despite numerous publications on SDHs, few report its potential risk factors in patients with SIH. In this study, we retrospectively investigated 93 consecutive SIH patients and divided them into an SDH group (n = 25) and a non-SDH (NSDH) group (n = 68). The clinical and radiographic characteristics of these 93 patients were analyzed, and then univariate analysis and further multiple logistic regression analysis were performed to identify the potential risk factors for the development of SDHs. The univariate analysis showed that advanced age, male gender, longer clinical course, dural enhancement, and the venous distension sign were associated with the development of SDHs. However, multivariate analysis only included the latter three factors. Our study reveals important radiological manifestations for predicting the development of SDHs in patients with SIH.

## Introduction

Spontaneous intracranial hypotension (SIH) is increasingly recognized as a noteworthy cause of orthostatic headache, resulting from a spontaneous cerebrospinal fluid (CSF) leak often associated with an underlying generalized connective tissue disorder [1–3]. Subdural haematoma (SDH) is a potentially life-threatening complication in patients with SIH, the incidence of which is reported to range from 20% to as high as 45% [1,4–10]. In serious cases, SIH patients with SDHs develop neurological deficits, decreased levels of consciousness, or even cerebral herniation [11]. And urgent neurosurgical drainage is required when the level of consciousness is decreased or the maximum thickness of the SDHs is >1 cm [12]. Although the rupture of bridging veins pulled away from the dura and brain descent due to low intracranial pressure have been widely proposed for the development of SDHs [1–3], the pathophysiology of SDHs in SIH remains unknown. Investigation of the risk factors for SDH may help to better understand its pathophysiology.

Despite numerous publications [6,13–16], studies on the risk factors for SDH in SIH patients are largely lacking. Lai et al. [5] reviewed 40 consecutive SIH patients with and without SDH in a case-controlled study and found that the presence of SDHs was associated with higher frequency of advanced age, male gender, the recurrence of severe headache, and neurological deficits. In a case series study, de Noronha et al. [6] found that SIH patients who presented with small subdural collections are prone to develop large SDHs. Nevertheless, as the overwhelming majority of the studies are case series or case reports without proper controls, the risk factors for SDH in SIH patients are still unclear.

In this study, we retrospectively reviewed 93 consecutive SIH patients in our hospital, of whom 25 had SDHs. The aim was to delineate the clinical and radiographic characteristics of SIH patients with SDH, and to further identify the potential risk factors for the development of SDHs.

## Materials and Methods

### Patients population

From January 2008 to March 2013, 93 SIH patients were admitted to our hospital. The diagnosis of SIH was made according to the criteria of the International Classification of Headache Disorder, 2nd edition (ICHD-2). Patients who had prior lumbar puncture, head trauma, epidural anesthesia, or other causes of CSF leaks were excluded. Each included patient received a cranial computed tomography (CT) scan on the day of admission. Subsequently, a brain magnetic resonance imaging (MRI) with gadolinium enhancement was performed in all patients. Then the 93 patients were divided into two groups, an SDH group (n = 25) and a non-SDH (NSDH) group (n = 68), according to the manifestations on CTs or MRIs. One or more SDHs were diagnosed if the subdural fluid showed hyperintensity on both T1- and T2-weighted images and isodensity or hyperdensity on CT scans [1,5,7,17–24]. There were 40 males and 53 females, with a mean age of 40.7 ± 9.4 years (range, 22 to 78). If an SDH was detected, further CT myelography was performed to identify the CSF leak sites and then a targeted epidural blood patch (EBP) was carried out. For those without SDHs, initial conservative treatment with bed rest and sufficient fluid intake was applied for at least 2 weeks, and if this failed, EBP treatment after CT myelography was carried out. Finally, 84 patients (25 SDH and 59 NSDH) received targeted EBP treatment, of whom 74 experienced considerable relief of symptoms after a single targeted EBP and the other 10 went into remission after more than one procedure. Nine patients with large haematomas (>1cm) had neurosurgical drainage of the SDHs as an emergency measure and subsequent EBP therapy. The Medical Ethics Committee of Sir Run Run Shaw Hospital, Zhejiang University School of Medicine, approved this study. All patients gave written informed consent as well as written permission for publication of their medical images.

### Clinical data

Age, sex, headache intensity, clinical course of SIH, CSF opening pressure, and coagulation indicators were recorded. The headache intensity was classified from mild to severe, according to the Numerical Rating Scale. For coagulation indicators, prothrombin time, activated partial thromoboplastin times, thrombin time, fibrinogen and platelet counts were used. For patients who received repeated lumbar punctures before diagnosis, the first CSF opening pressure was recorded.

### Radiographic findings

**Fig 1:**
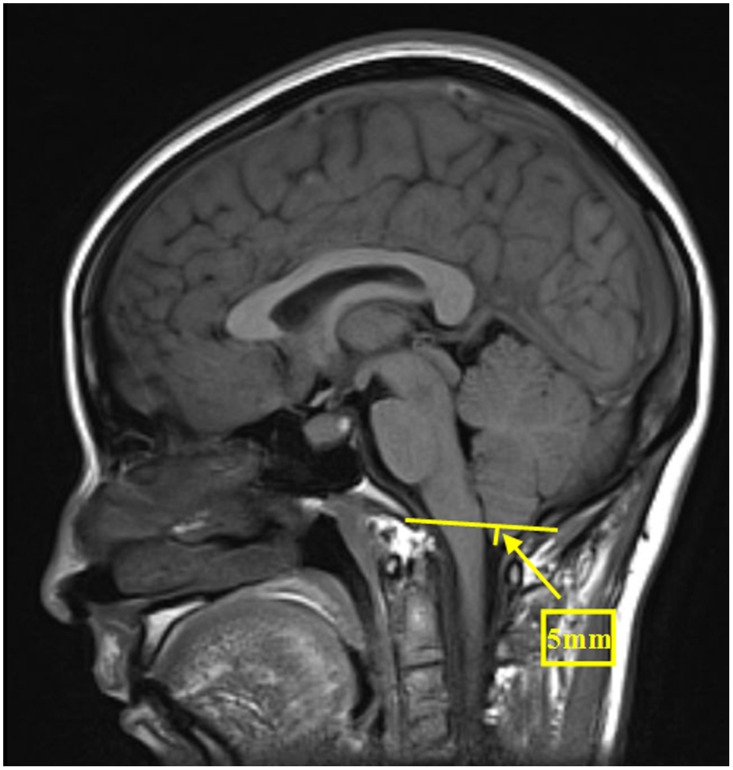
Typical MRI manifestation of brain sagging. Midsagittal T1-weighted MRI shows downward displacement of the cerebellar tonsil by 5 mm (arrow).

**Fig 2:**
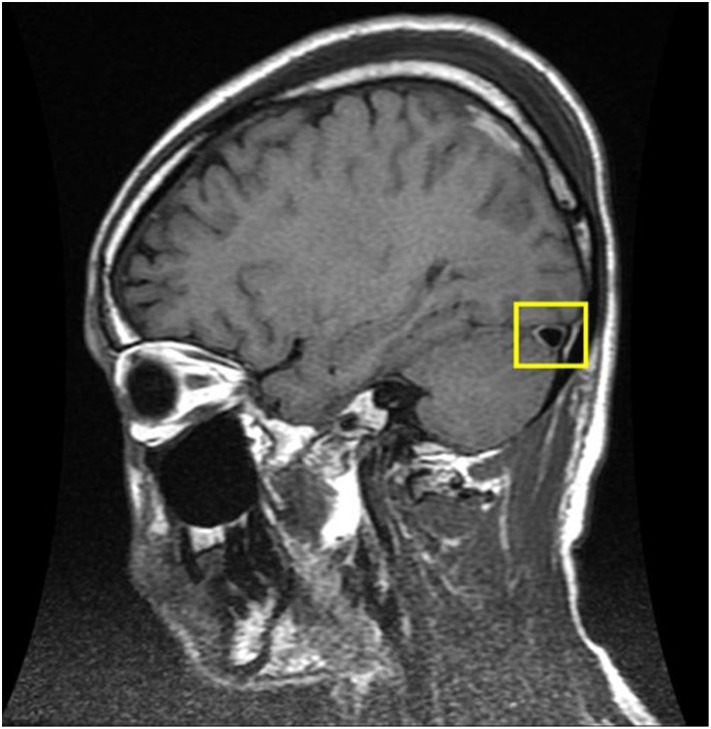
Typical MRI manifestation of the venous distension sign. T1-weighted MRI through the midportion of the dominant transverse sinus shows the venous distension sign (box).

**Fig 3:**
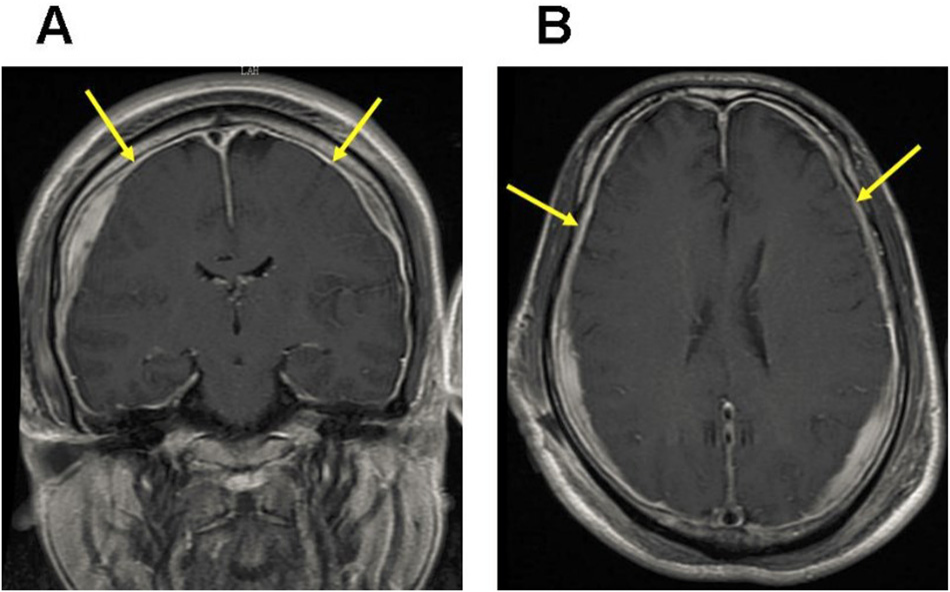
Typical MRI manifestation of dural enhancement. T1-weighted MRIs with gadolinium enhancement shows dural enhancement in coronal (A) and horizontal (B) images (arrows).

The brain MRI was performed using a 1.5-T System (Siemens, Germany) with a head-sense-coil. Midsagittal T1-weighted MRIs were selected for the evaluation of brain sagging, which was defined as either cerebral aqueduct displacement ≥1.8 mm or cerebellar tonsil displacement ≥4.3 mm (Fig 1) [25]. The midportion of the dominant transverse sinus on sagittal T1-weighted MRIs was used for assessing the venous distension sign (VDS), as proposed by Farb et al. (Fig 2) [26]. Dural enhancement was evaluated on axial and coronal T1-weighted images with gadolinium enhancement (Fig 3).

**Fig 4:**
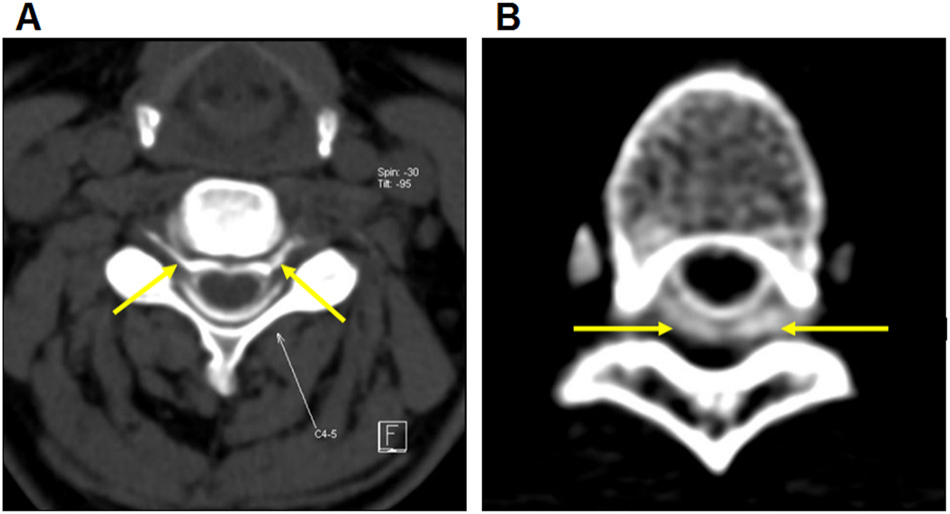
CT myelography for evaluating dural leakage. CT myelography shows contrast tracking along bilateral nerve roots (A, arrows) and collections of contrast within the paraspinal soft tissue (B, arrows). Images taken at segments C4-5 (A) and T5-6 (B).

CT myelography for detecting the sites of dural leakage was performed on a second-generation dual-source CT with tube voltages set at 100 kVp and 140 kVp (with tin filter). Leakage varied from a small amount of contrast tracking along a single nerve root to extensive bilateral collections of contrast within the paraspinal soft tissues (Fig 4) [7].

Two neuroradiologists (Q.Z. and D.W.) blinded to the study design reviewed all images to document the presence or absence of SDHs, and evaluated pachymeningeal enhancement, VDS, brain sagging, and the number of dural leaks. Inter-observer repeatability was calculated using the intra-class correlation coefficient, which ranged from 0.86 to 0.92 for the MRI evaluations and was 0.81 for the CT assessment, indicating good inter-observer reliability.

### Statistical Analysis

Statistical analyses were performed using SPSS 19.0 software. In univariate analysis, Student's *t* test was used for comparisons of continuous variables and the *χ* ^2^ test for comparisons of categorical variables. Variables were subjected to multivariate analysis with a logistic regression and forward stepwise selection after univariate analysis. The presence or absence of SDH (coded as 1 or 0, respectively) was set as the independent variable. An unconditional multiple logistic regression model was used to calculate the odds ratio (OR) and 95% confidence interval (CI). *P* <0.05 indicated statistical significance.

## Results

Twenty-five patients developed SDHs (16 males and 9 females; average age 43.9 ± 12.0; range, 22 to 78 years). Of these, 17 experienced bilateral haematomas and 8 developed unilateral haematomas. There were 68 NSDH patients (24 males and 44 females; average age 39.4 ± 7.7; range, 25 to 57 years).

We first performed univariate analysis to identify the potential risk factors for SDH (Table 1), and found that advanced age (P = 0.043), male gender (P = 0.032), longer clinical course (P = 0.012), dural enhancement (P <0.001) and VDS (P <0.001) were associated with the presence of SDHs. We did not find any differences in the intensity of headache, CSF pressure, coagulation markers, or platelet counts between the SDH and NSDH groups. Nor did we find any differences in brain sagging or the number of CSF leaks between the two groups.

**Table 1:** Results of univariate analysis.

Variables	SDH (n = 25)	NSDH (n = 68)	P value
Age (years)	43.9 ± 12.0	39.4 ± 7.7	0.043
Gender (M/F)	16/9	24/44	0.018
Severe headache	21	49	0.411
Clinical course (days)	43.1 ± 29.7	26.9 ± 24.9	0.012
CSF opening pressure (mmH_2_O)	41.8 ± 23.9	47.5 ± 38.0	0.409
PT (s)	13.2 ± 0.6	13.1 ± 0.6	0.382
APTT (s)	34.2 ± 3.4	33.2 ± 3.8	0.261
TT (s)	16.3 ± 1.6	16.2 ± 0.7	0.164
FG (s)	2.8 ± 0.8	2.8 ± 0.6	0.139
PLT (×10^12^/L)	184.0 ± 97.4	200.6 ± 55.2	0.344
Dural enhancement	24	34	0.000
VDS	23	25	0.000
Brain descent	8	12	0.159
Number of leaks	7.8 ± 5.1	6.9 ± 4.0	0.432

APTT, activated partial thromoboplastin times; CSF, cerebrospinal fluid; F, female; FG, fibrinogen; M, male; NSDH, non-subdural haematoma; PLT, platelets; PT, prothrombin time; SDH, subdural haematoma; TT, thrombin time; VDS, venous distension sign.

Multivariate logistic regression was calculated for the five identified variables. Clinical course duration was still associated with the development of SDHs in patients with SIH (OR = 1.055, 95% CI: 1.016–1.095, P = 0.005), as well as dural enhancement (OR = 26.026, 95% CI: 1.158–584.864, P = 0.04) and VDS (OR = 22.102, 95% CI: 1.38–205.253, P = 0.006), but age and sex were no longer significant factors (Table 2).

**Table 2:** Results of multivariate analysis.

Variables	Odds Ratio	95% CI for OR	*P* value
Clinical course	1.055	1.016–1.095	0.005
Dural enhancement	26.026	1.158–584.864	0.040
VDS	22.102	1.38–205.253	0.006

OR, odds ratio; VDS, venous distension sign.

## Discussion

Our main finding was that the clinical course duration, dural enhancement, and VDS were correlated with the development of SDHs in patients with SIH.

According to de Noronha et al. [6], published reports are unclear about the pathological nature of "collection", "effusion", “hygroma" and "haematoma", and these terms are used interchangeably. And authors themselves use the term "collection" in all their cases except when blood is identified at surgery. When the operative findings are not available for each case, imaging diagnosis is the most commonly-used method for determining the presence or absence of SDHs in SIH [1,5,7]. We adopted the imaging diagnosis criteria used by most investigators, as noted above [1,5,7,17–24]. Non-hemorrhagic subdural collections show hypo- or isointensity on T1-weighted images, and hyperintensity on T2-weighted MRIs [1, 7]. Furthermore, blood was identified in all 9 patients with large hematomas and undergoing neurosurgical drainage of SDHs as an emergency measure,. That, in turn, confirmed the effectiveness and validity of our imaging diagnosis of SDHs in SIH. And the incidence of SDHs in SIH our cohort was 26.9%, in agreement with reported data [5,7–10].

Age and sex were not identified as risk factors in this study. Generally, advanced age and male gender are considered to be correlated with chronic SDHs [16,27]. Older people are thought to have a higher tendency to suffer an SDH due to brain atrophy, which would enlarge the subarachnoid space and stretch the bridging veins [16,28]. And it has been reported that the male preponderance of chronic SDHs may be due to greater exposure to head injuries [15,16,27]. In patients with SIH, however, only Lai et al. [5] have investigated the association between these two factors and SDHs, and they reached conclusions different from ours. The discrepancies are probably because they only conducted univariate without further multivariate analysis.

The present study showed that the clinical course was longer in the SDH group than in the NSDH group. Our prior investigation found that the incidence of SDHs is higher in patients with SIH of long duration (≥30 days) than those of short duration (<30 days) (50% *versus* 11.8%, P = 0.018) [27]. We postulated that the longer duration would lead to persistent compensation by venous engorgement, according to the Monor-Kellie doctrine. As a result, the bridging veins are pulled away from the dura. Once the dural vessels are excessively dilated, they rupture and a SDH occurs.

The CSF opening pressure showed no statistical differences between the two groups. Different investigations give different results as to whether intracranial pressure is increased in the presence of SDHs in SIH patients. Some authors postulate that the development of SDHs corrects the abnormally low intracranial pressure or volume [1]. Sato et al. [20] reported an SIH case with an initial CSF opening pressure of 40 mm H_2_O, and the development of SDHs during follow-up elevated the value to 170 mm H_2_O. However, many studies have shown that the development of SDHs does not necessarily elevate the intracranial pressure. Schievink et al. [23] described 3 cases of SDHs caused by spontaneous spinal CSF leaks, and all the CSF opening pressures were below normal. Nakajima et al. [29] reported a case of bilateral SDHs associated with SIH, and all percutaneous aspirations revealed negative or low intracranial pressure. We recently reported four cases of SDHs in patients with SIH, and revealed that three out of the four patients had low CSF opening pressure [24]. Therefore, we think that the presence of SDHs in SIH is not necessarily correlated with an elevated or normal intracranial pressure. Apart from the presence or absence of SDHs, we speculate that other factors, such as the characteristics of the SDHs and factors related to SIH, may have significant effects on the intracranial pressure in patients with SIH. However, the exact mechanisms are not yet known.

Many studies have investigated the association between spontaneous chronic SDHs and changes in coagulation parameters [16,30–35]. A coagulation disorder should be suspected when an unexplained hemorrhage occurs, especially in young patients [33]. Recent reports have shown that anti-platelet therapy might increase the risk of intracranial hemorrhage, including chronic SDHs, by inhibiting platelet function [16,34,35]. However, we did not find any differences in the coagulation parameters between the SDH and NSDH groups. We surmised that mechanical stress rather than potential coagulation disorders is more likely to be responsible for the occurrence of SDHs in patients with SIH.

In addition to SDHs, the most marked MRI manifestations of SIH were diffuse pachymeningeal enhancement, engorgement of venous structures, and brain sagging. In patients with SIH, the loss of CSF volume can lead to a compensatory increase in the intracranial vascular component, which accounts for the occurrence of pachymeningeal enhancement and the engorgement of venous structures [17]. The sagging of the brain is caused by the decrease of CSF buoyancy due to the loss of CSF volume and can be exacerbated by subdural effusions or haematomas [17,36].

The pachymeningeal enhancement and the engorgement of venous structures differed significantly between the two groups, but not brain sagging. A continuous thickening of the dura mater accompanied by a diffuse and intense contrast enhancement was observed in all but one of the patients in the SDH group but only 34 of 68 patients in the NSDH group (P = 0.04). VDS is a highly sensitive and accurate sign for assessing the presence of intracranial venous engorgement in SIH patients [26]. This can easily be evaluated on non-enhanced sagittal T1-weighted images. In this study, VDS was present in 23 out of the 25 patients in the SDH group, compared to 25 of 68 in the NSDH group (P = 0.006). Thus, we speculated that CSF volume depletion was more rapid or continuous in the SDH group than in the NSDH group, which induced relatively more rapid and stronger compensatory accommodation by the accumulation of blood and distension of the venous sinuses. The compensatory vascular engorgement and increased flow leads to pachymeningeal hyperemia, enhancement, and a positive VDS [37].

Brain sagging appeared to be more common in the SDH group but the difference was not statistically significant. Interestingly, we found that the incidence of brain sagging was much lower than those of the other two MRI signs in both groups. The reasons may be as follows: 1) the supine position of the patients during MRI examination may lead to underestimation of the incidence of brain descent [38]; and 2) since sagging is partly caused by downward force due to subdural effusions or haematomas, it may occur until these mechanisms have been exhausted [38].

Spontaneous spinal CSF leaks caused by structural weakness of the spinal meninges are well recognized as the underlying etiology of SIH [7]. CT myelography is the study of choice to accurately locate the sites of leaks in patients with SIH. Our study revealed that the average leak sites in the SDH group was 7.8 (range, 1 to 11), compared to 6.9 in the NSDH group (range, 1 to 10), with no significant difference. Only 3 out of the 84 patients had a single leak site, in accord with the findings of our previous studies [39]. We failed to find any correlation between the number of leaks and SDH in SIH patients, probably because the size of the leak site and the velocity of leakage rather than the number of leaks are directly correlated with SIH. Furthermore, the time delay between intrathecal contrast administration and CT myelography might allow large amounts of epidural fluid to extravasate in cases with a high-volume CSF leak. Consequently, the leak sites might be obscured. Dynamic sequential DynaCT myelograms could be superior in demonstrating the “real” sites of leakage. This would be a valuable adjunct in diagnosis in future work [40].

This study had some limitations. First, in patients with "asymptomatic" SDHs, the interval between the onset of the symptoms of SIH and the development of SDHs may have been overestimated. This could predispose to a false association between the course and the development of SDHs. However, the proportion of "asymptomatic" SDHs in our study was relatively low (24%, 6/25), and the CT scans for detecting SDHs were performed relatively promptly. Therefore, we suppose that these "asymptomatic" SDHs probably did not affect our results. Second, since SIH is not a common disease, the sample size of SDHs in this study was small. Future studies with larger sample are needed.
